# tsRNA-00764 Regulates Estrogen and Progesterone Synthesis and Lipid Deposition by Targeting PPAR-γ in Duck Granulosa Cells

**DOI:** 10.3390/ijms252011251

**Published:** 2024-10-19

**Authors:** Yaru Chen, Yan Wu, Jinsong Pi, Ming Fu, Jie Shen, Hao Zhang, Jinping Du

**Affiliations:** 1 Institute of Animal Science and Veterinary Medicine, Hubei Academy of Agricultural Sciences, Wuhan 430064, China; chenyaru@hbaas.com (Y.C.); fuming19911203@163.com (M.F.); 15927657060@163.com (J.S.); 15172520011@163.com (H.Z.); ddjinpin@163.com (J.D.); 2 Hubei Key Laboratory of Animal Embryo Engineering and Molecular Breeding, Wuhan 430064, China

**Keywords:** transfer RNA-derived small RNAs (tsRNAs), follicle selection, duck granulosa cells, progesterone synthesis, estrogen synthesis, lipid deposition

## Abstract

Transfer RNA-derived small RNAs (tsRNAs) are novel regulatory small non-coding RNAs that have been found to modulate many life activities in recent years. However, the exact functions of tsRNAs in follicle development remain unclear. Follicle development is a remarkably complex process that follows a strict hierarchy and is strongly associated with reproductive performance in ducks. The process of converting small yellow follicles into hierarchal follicles is known as follicle selection, which directly determines the number of mature follicles. We performed small RNA sequencing during follicle selection in ducks and identified tsRNA-00764 as the target of interest based on tsRNA expression profiles in this study. Bioinformatics analyses and luciferase reporter assays further revealed that peroxisome proliferator-activated receptor-γ (PPAR-γ) was the target gene of tsRNA-00764. Moreover, tsRNA-00764 knockdown promoted estrogen and progesterone synthesis and lipid deposition in duck granulosa cells, while a PPAR-γ inhibitor reversed the above phenomenon. Taken together, these results demonstrate that tsRNA-00764, differentially expressed in pre-hierarchal and hierarchy follicles, modulates estrogen and progesterone synthesis and lipid deposition by targeting PPAR-γ in duck granulosa cells, serving as a potential novel mechanism of follicle selection. Overall, our findings provide a theoretical foundation for further exploration of the molecular mechanisms underlying follicle development and production performance in ducks.

## 1. Introduction

The ovary is an important reproductive organ which is responsible for follicular development and hormone secretion in poultry [1]. Ovarian follicles serve as basic and non-renewable reproductive units, consisting of an oocyte and surrounding granulosa and theca cells [2]. Follicle development in poultry mainly involves quiescent primordial follicles, slow-growing pre-hierarchal follicles, and hierarchical follicle stages. The process of selecting an optimal follicle from small yellow follicles for development into hierarchal follicles is called follicle selection [3]. Follicle selection determines the number of mature follicles and ultimately affects reproductive performance [4].

Follicle selection is a remarkably complex, carefully orchestrated process that requires constant communication between the oocyte and its neighboring granulosa cells [5]. Granulosa cells, as an important part of the follicle, are responsible for maintaining the follicle’s formation [6]. Granulosa cell differentiation and steroid hormone secretion are the hallmark characteristics of follicle selection in poultry, along with the elevated expression of steroid-related genes, such as cytochrome P450 family 19 subfamily A member 1 (CYP19A1) and steroidogenic acute regulatory protein (STAR) [7]. Steroid hormones are derived from cholesterol synthesis and are modulated by lipid metabolism. Peroxisome proliferator-activated receptor-γ (PPAR-γ), ATP-citrate lyase (ACLY), stearoyl-CoA desaturase (SCD), and fatty acid synthase (FASN) are important enzymes in the de novo lipogenesis pathway and participate in the regulation of follicular development in humans, mice, bovines, chickens, and geese [8,9,10].

There are significant differences between pre-hierarchal and hierarchical follicles, and several studies have demonstrated that non-coding RNAs, including microRNAs (miRNAs), long non-coding RNAs (lncRNAs), and circular RNAs (circRNAs), are involved in follicle selection [11,12,13]. For instance, miR-22-3p targets phosphatase tensin homolog (PTEN) to activate the PI3K/Akt/mTOR pathway in chicken hierarchical follicles, thereby promoting granulosa cell proliferation, steroid hormone secretion, and lipid accumulation [14]. Long non-coding RNA13814 promotes the apoptosis of duck granulosa cells by binding to apla-mir-145-4 and enhances DNA damage-inducible transcript 3 (DDIT3) expression, leading to the prevention of the development of hierarchical follicles [15]. The low expression of circRALGPS2-212aa inhibits follicle atresia and promotes pre-hierarchal development in chickens by forming a complex with poly ADP-ribose polymerase 1 (PARP1) and high mobility group box 1 (HMGB1) [16]. tRNA-derived small RNAs (tsRNAs) are newly discovered small non-coding RNAs that derive from tRNAs precursors or mature tRNAs [17]. Notably, several studies have revealed that tsRNAs have similar mechanisms to miRNAs, which bind to the 3′-UTR of mRNA and regulate its expression at the post-transcriptional level [18,19]. Furthermore, tsRNAs participate in multiple biological processes, such as oncologic disorders, stress responses, fat deposition, spermatogenesis, and embryonic development [18]. However, the exact roles of tsRNAs in follicle development remain unclear.

In this study, we compared the tsRNA expression accumulation profiles in duck granulosa cells of pre-hierarchal and hierarchical follicles using high-throughput RNA-seq. Moreover, we demonstrated that tsRNA-00764 suppressed estrogen and progesterone synthesis and lipid deposition by targeting PPAR-γ in duck granulosa cells, which could block the transition from pre-hierarchal follicles to hierarchical follicles. Our work aims to confirm the molecular mechanism by which differentially expressed tsRNAs modulate follicle selection and provide a novel insight into the regulation of production performance in ducks.

## 2. Results

### 2.1. Accumulation Characteristics of tsRNAs in Duck Follicles

**Figure 1:**
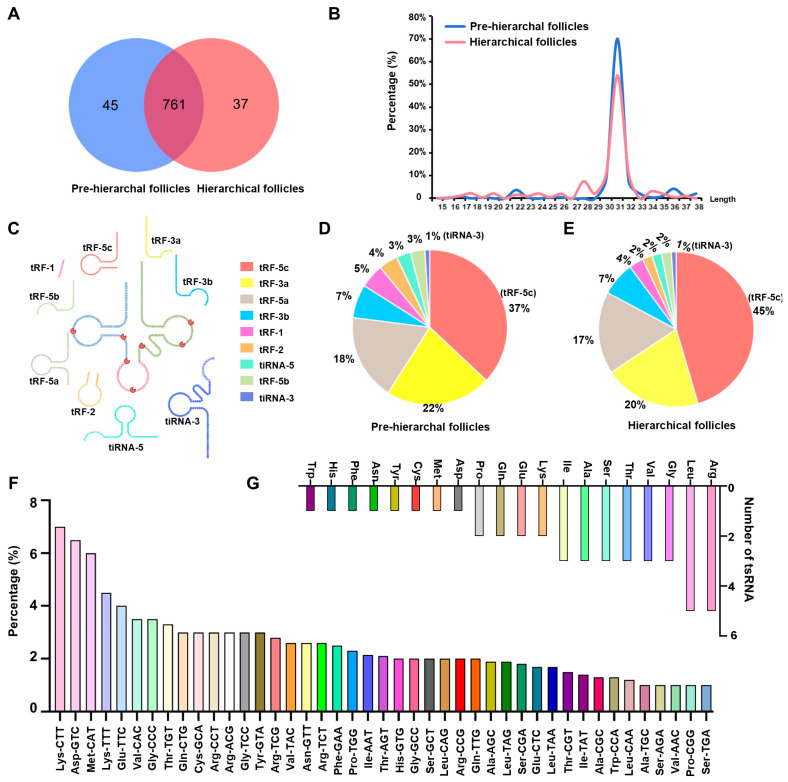
The type and source of tsRNAs in duck follicles. (**A**) A Venn diagram showing the number of tsRNAs expressed in pre-hierarchal and hierarchical follicles; (**B**) the length distribution of tsRNAs in pre-hierarchal and hierarchical follicles; (**C**) a schematic diagram of tsRNA classifications; (**D**,**E**) the percentage of each type of tsRNA in pre-hierarchal (**D**) and hierarchical (**E**) follicles; (**F**) the source of tsRNAs in pre-hierarchal and hierarchical follicles; (**G**) the number and species of tRNAs that generated tsRNAs.

A total of 843 tsRNAs, mainly 27–32 nucleotides in length, were identified in pre-hierarchal and hierarchical follicles based on the duck genomic sequence using high-throughput sequencing (Figure 1A,B). Among nine different tsRNA subtypes, the percentage of tRF-5c was the highest, but the percentage of tiRNA-3 was lowest in both pre-hierarchal and hierarchical follicles (Figure 1C–E). Indeed, these tsRNAs were obtained from 44 tRNA subtypes, of which tRNA-Lys-CTT (6.70%) generated the most tsRNAs (Figure 1F). Analysis of the different types of tsRNAs revealed that 44 tRNA isoforms were aminoacylated by 20 amino acids, with 5 each of tRNA-Arg and tRNA-Leu, 3 each of tRNA-Gly, tRNA-Val, tRNA-Thr, tRNA-Ser, tRNA-Ala, and tRNA-ILe, 2 each of tRNA-Lys, tRNA-Glu, tRNA-Gln, and tRNA-Pro, and 1 each of tRNA-Cys, tRNA-Met, tRNA-Asp, tRNA-Tyr, tRNA-Asn, tRNA-Phe, tRNA-His, and tRNA-Trp (Figure 1G).

### 2.2. Differentially Expressed tsRNAs and mRNAs Between Pre-Hierarchal and Hierarchical Follicles

**Figure 2:**
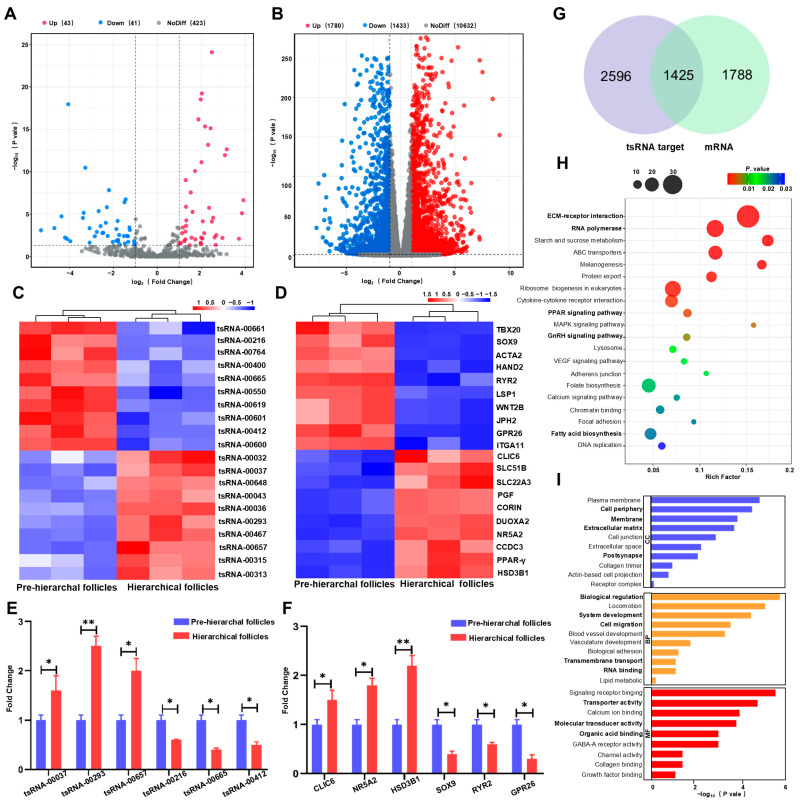
Analysis of differentially expressed tsRNAs and mRNAs. (**A**,**B**) A volcano plot showing the differentially expressed tsRNAs (**A**) and mRNAs (**B**) between pre-hierarchal and hierarchical follicles; (**C**,**D**) the heat map reveals the top 20 differentially expressed tsRNAs (**C**) and mRNAs (**D**) between pre-hierarchal and hierarchical follicles; (**E**,**F**) validation of differentially expressed tsRNAs (**E**) and mRNAs (**F**) using RT-qPCR between pre-hierarchal and hierarchical follicles; (**G**) a Venn map of the top 10 most highly differentially expressed tsRNA targets and differentially expressed mRNAs; (**H**,**I**) KEGG (**H**) and GO (**I**) analysis of the overlapping genes in (**G**). * *p* < 0.05 and ** *p* < 0.01.

We identified 84 tsRNAs that were differentially expressed between pre-hierarchal and hierarchical follicles, with 43 up-regulated and 41 down-regulated (|log_2_Fc| > 1 and *p* < 0.05) (Figure 2A, Table S1). Additionally, there were 1780 mRNAs up-regulated and 1433 mRNAs down-regulated in hierarchical follicles (|log_2_Fc| > 1 and *p* < 0.05) (Figure 2B, Table S2). The 20 most significantly differentially expressed tsRNAs and mRNAs are shown in the heatmap (Figure 2C,D), and 6 randomly selected tsRNAs and mRNAs were validated using quantitative RT-PCR (Figure 2E,F). We identified 1425 overlapping expressed genes in the targets of the top 10 most highly differentially expressed tsRNAs and differentially expressed mRNAs through overlap analysis (Figure 2G). A Kyoto Encyclopedia of Genes and Genomes (KEGG) analysis demonstrated that overlapping genes were mostly enriched in ECM–receptor interaction, RNA polymerase, PPAR signaling pathway, GnRH signaling pathway, and fatty acid biosynthesis (Figure 2H). A Gene Ontology (GO) analysis showed that the overlapping genes were divided into different categories, including biological regulation, system development, cell migration, transmembrane transport, and RNA binding for biological processes; cell periphery, membrane, extracellular matrix, and postsynapse for cellular components; and transporter, molecular transducer activity, and organic acid binding for molecular functions (Figure 2I).

### 2.3. PPAR-γ Is the Target Gene of tsRNA-00764

**Figure 3:**
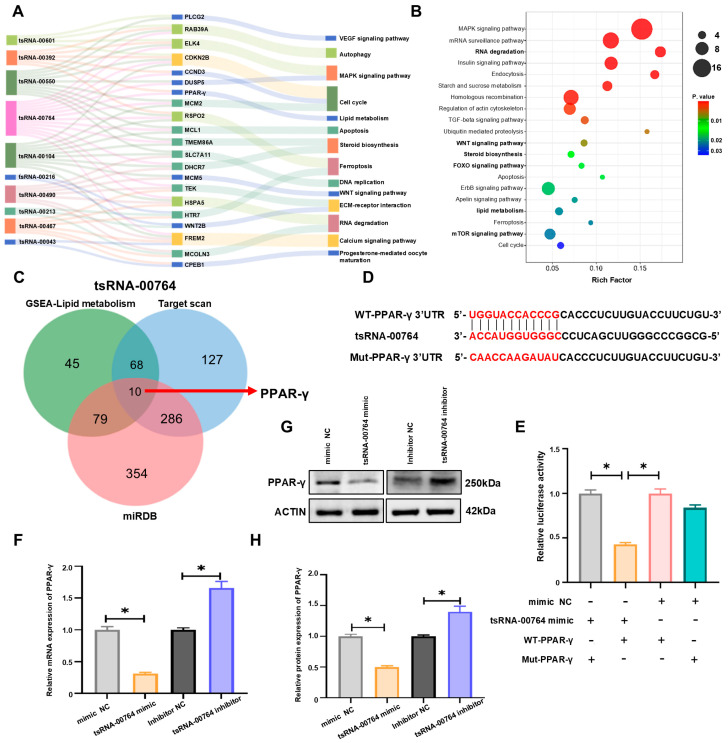
PPAR-γ is the target gene of tsRNA-00764. (**A**) An alluvial diagram of 10 differentially expressed tsRNAs, their target genes, and the GO terms; (**B**) the top 20 enriched KEGG pathways of tsRNA-00764 target genes; (**C**) a Venn diagram of the target genes of tsRNA-00764 in the Target scan, miRDB, and GSEA lipid metabolism; (**D**) the predicted binding site between PPAR-γ and tsRNA-00764, and the red text represents binding sites of the tsRNA-00764 and PPAR-γ interaction network; (**E**) the targeting relationship between PPAR-γ and tsRNA-00764 was measured by a dual-luciferase assay; (**F**) the PPAR-γ mRNA expression level in duck granulosa cells after treatment with the tsRNA-00764 mimic or the tsRNA-00764 inhibitor; (**G**) the PPAR-γ protein expression level in duck granulosa cells after treatment with the tsRNA-00764 mimic or the tsRNA-00764 inhibitor; (**H**) the quantification of the PPAR-γ protein level in (**G**). * *p* < 0.05.

We selected 10 tsRNAs with common target genes from the differentially expressed tsRNAs and constructed a tsRNA-mRNA-GO term interaction network to explore their functions in follicle development (Figure 3A). The expression of tsRNA-00764 was elevated in pre-hierarchal follicles compared with hierarchical follicles. To investigate the underlying mechanism of follicle selection, the potential targets of tsRNA-00764 were predicted using target gene prediction software (TargetScan (https://www.targetscan.org/ accessed on 29 January 2024) and miRDB (http://www.microrna.org/microrna/home.do accessed on 29 January 2024)). The KEGG analysis revealed that the target genes were enriched in RNA degradation, lipid metabolism, and steroid hormone biosynthesis, including mTOR, WNT, and FOXO signaling (Figure 3B). We found that 202 genes associated with lipid metabolism processes were enriched via geneset enrichment analysis (GSEA) (Table S3). The Venn diagrams revealed 10 genes including PPAR-γ in the GSEA lipid metabolism, miRDB, and TargetScan results (Figure 3C). Furthermore, bioinformatics software showed the presence of binding sites between tsRNA-00764 and PPAR-γ (Figure 3D). We constructed wild-type (WT) and mutant (Mut) PPAR-γ luciferase reporter plasmids based on the predicted target binding sites. The luciferase reporter assays indicated that the tsRNA-00764 mimic decreased WT-PPAR-γ 3′UTR activity but had no effect on Mut-PPAR-γ 3′UTR, indicating that tsRNA-00764 bound to the PPAR-γ 3′UTR (* *p* < 0.05, Figure 3E). The results demonstrate that the tsRNA-00764 mimic significantly inhibited PPAR-γ expression, while the tsRNA-00764 inhibitor displayed the opposite effects (* *p* < 0.05, Figure 3F–H). Collectively, our results reveal that PPAR-γ is the target gene of tsRNA-00764.

### 2.4. tsRNA-00764 Suppresses Estrogen and Progesterone Synthesis and Lipid Deposition in Duck Granulosa Cells

**Figure 4:**
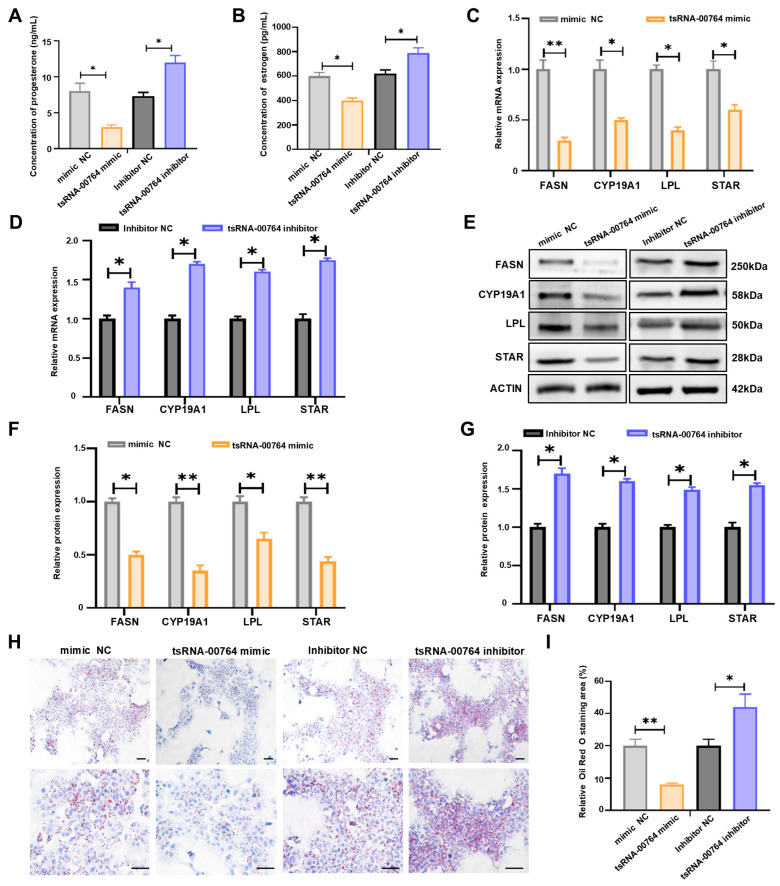
tsRNA-00764 inhibits estrogen and progesterone synthesis and lipid deposition in duck granulosa cells. (**A**,**B**) The concentration of progesterone (**A**) and estrogen (**B**) in duck granulosa cells after treatment with the tsRNA-00764 mimic or the tsRNA-00764 inhibitor; (**C**,**D**) the FASN, CYP19A1, LPL, and STAR mRNA expression levels were detected by RT-qPCR in duck granulosa cells transfected with the tsRNA-00764 mimic (**C**) or the tsRNA-00764 inhibitor (**D**); (**E**) the FASN, CYP19A1, LPL, and STAR protein expression levels were detected by Western blot in duck granulosa cells transfected with the tsRNA-00764 mimic or the tsRNA-00764 inhibitor; (**F**,**G**) the quantification of the FASN, CYP19A1, LPL, and STAR protein levels in (**E**); (**H**) the accumulated lipid droplet was measured by Oil Red O staining in duck granulosa cells transfected with the tsRNA-00764 mimic or the tsRNA-00764 inhibitor (*n* = 4). Scale bar: 30 μm. (**I**) The quantification of the accumulated lipid droplet in (**H**). * *p* < 0.05 and ** *p* < 0.01.

To further identify the functions of tsRNA-00764 in follicle development, we examined estrogen and progesterone levels in duck granulosa cells after transfection with the tsRNA-00764 mimic and inhibitor. We found that the concentration of estrogen and progesterone was inhibited by the tsRNA-00764 mimic and promoted by the tsRNA-00764 inhibitor (* *p* < 0.05, Figure 4A,B). To further explore the regulation of tsRNA-00764 in hormone synthesis, we examined the expressions of steroid hormone synthesis-associated genes, including cytochrome P450 family 19 subfamily A member 1 (CYP19A1) and steroidogenic acute regulatory protein (STAR), using quantitative RT-PCR and Western blot in duck granulosa cells. Indeed, tsRNA-00764 inhibited the expression of steroidogenic-related genes (STAR and CYP19A1), suggesting that tsRNA-00764 suppressed steroid hormone synthesis in duck granulosa cells (* *p* < 0.05, ** *p* < 0.01, Figure 4C–G). Oil Red O staining demonstrated that the tsRNA-00764 mimic reduced lipid droplet accumulation, whereas the tsRNA-00764 inhibitor promoted lipid droplet accumulation in duck granulosa cells (* *p* < 0.05, ** *p* < 0.01, Figure 4H,I). The expression of lipid deposition-related genes, including fatty acid synthase (FASN) and lipoprotein lipase (LPL), was decreased after transfection with the tsRNA-00764 mimic, while the tsRNA-00764 inhibitor promoted the expression of these genes (* *p* < 0.05, ** *p* < 0.01, Figure 4C–G). Thus, we concluded that tsRNA-00764 represses estrogen and progesterone synthesis and lipid deposition in duck granulosa cells.

### 2.5. tsRNA-00764 Modulates Estrogen and Progesterone Synthesis and Lipid Deposition by Targeting PPAR-γ in Duck Granulosa Cells

**Figure 5:**
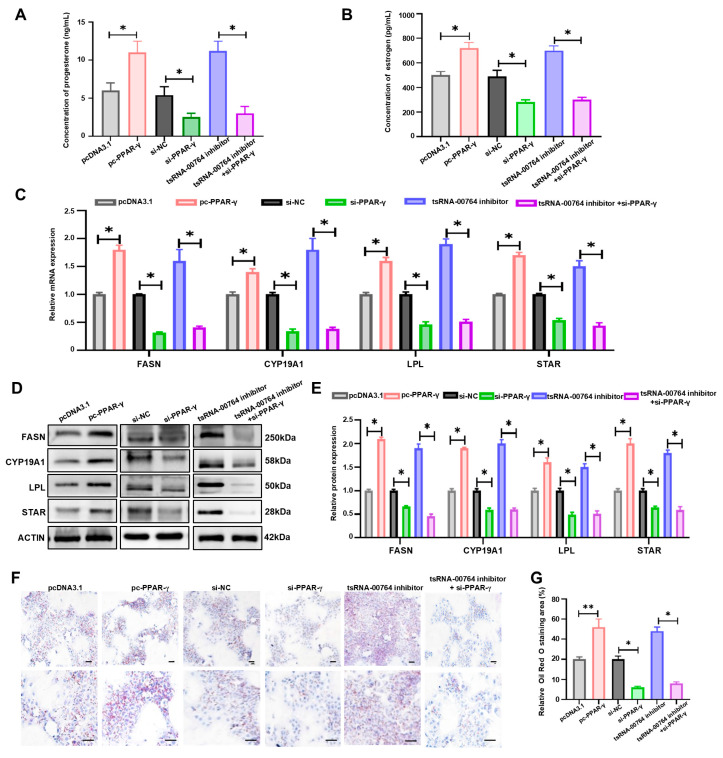
tsRNA-00764 regulates estrogen and progesterone synthesis and lipid deposition via the PPAR-γ pathway in duck granulosa cells. (**A**,**B**) The concentration of progesterone (**A**) and estrogen (**B**) in pcDNA3.1-PPAR-γ-treated duck granulosa cells, PPAR-γ siRNA-treated duck granulosa cells, tsRNA-00764 inhibitor-treated duck granulosa cells, and tsRNA-00764 inhibitor + PPAR-γ siRNA co-treated duck granulosa cells; (**C**) the FASN, CYP19A1, LPL, and STAR mRNA expression level was detected by RT-qPCR in pcDNA3.1-PPAR-γ-treated duck granulosa cells, PPAR-γ siRNA-treated duck granulosa cells, tsRNA-00764 inhibitor-treated duck granulosa cells, and tsRNA-00764 inhibitor + PPAR-γ siRNA co-treated duck granulosa cells; (**D**) the FASN, CYP19A1, LPL, and STAR protein expression level was detected by Western blot in pcDNA3.1-PPAR-γ-treated duck granulosa cells, PPAR-γ siRNA-treated duck granulosa cells, tsRNA-00764 inhibitor-treated duck granulosa cells, and tsRNA-00764 inhibitor + PPAR-γ siRNA co-treated duck granulosa cells; (**E**) the quantification of the FASN, CYP19A1, LPL, and STAR protein level in (**D**); (**F**) the accumulated lipid droplet was measured by Oil Red O staining in pcDNA3.1-PPAR-γ-treated duck granulosa cells, PPAR-γ siRNA-treated duck granulosa cells, tsRNA-00764 inhibitor-treated duck granulosa cells, and tsRNA-00764 inhibitor + PPAR-γ siRNA co-treated duck granulosa cells (*n* = 4). Scale bar: 30 μm. (**G**) The quantification of the accumulated lipid droplet in (**F**). * *p* < 0.05 and ** *p* < 0.01.

To investigate the functions of PPAR-γ, the PPAR-γ-overexpressed vector (pcDNA3.1-PPAR-γ) and si-PPAR-γ were constructed and transfected into duck granulosa cells. The concentration of progesterone and estrogen was elevated in PPAR-γ-overexpressed granulosa cells (pc-PPAR-γ) but was significantly reduced in si-PPAR-γ-treated cells (si-PPAR-γ) (* *p* < 0.05, Figure 5A,B). PPAR-γ promoted the mRNA and protein level of steroidogenic-related genes (STAR and CYP19A1) (* *p* < 0.05, Figure 5C–E). PPAR-γ knockdown prevented the improvement in the estrogen and progesterone levels and the enhancement of steroidogenic-related gene (STAR and CYP19A1) expression in tsRNA-00764 inhibitor-treated cells (* *p* < 0.05, Figure 5A–E). In addition, PPAR-γ overexpression promoted the expression of lipid deposition-related genes (FASN and LPL) (* *p* < 0.05, Figure 5C–E) and accelerated lipid droplet accumulation in duck granulosa cells (* *p* < 0.05, ** *p* < 0.01, Figure 5F–G), whereas the opposite results were found in si-PPAR-γ-treated cells. The promotion of lipid droplet accumulation and lipid deposition-related gene (FASN and LPL) expression by the tsRNA-00764 inhibitor was reversed after co-transfection with si-PPAR-γ (* *p* < 0.05, ** *p* < 0.01, Figure 5F–G). These findings demonstrate that tsRNA-00764 suppresses estrogen and progesterone synthesis and lipid deposition by regulating PPAR-γ in duck granulosa cells.

## 3. Discussion

Follicle selection is a critical stage of follicle development in laying ducks which is closely associated with production performance and fecundity [20]. Indeed, follicle development follows a strict hierarchy in laying ducks [21]. Follicles rarely degenerate after being selected for ovulation, and thus, the molecular mechanisms of development from pre-hierarchal to hierarchical follicles have always been a research hotspot [22]. Multiple studies have reported that non-coding RNAs, such as miRNAs, lncRNAs, and circRNAs, are involved in follicle selection in poultry. tRNA-derived small RNAs (tsRNAs) are novel regulatory non-coding small RNAs that participate in various physiological processes, including oncologic disorders, stress responses, fat deposition, spermatogenesis, and embryonic development [23,24,25]. Here, the exact roles of tsRNAs in follicle development remain unclear. Therefore, we investigated the characteristics and functions of tsRNAs and mRNAs in pre-hierarchal and hierarchical follicles, which may contribute to a better understanding of the mechanisms of follicle development in ducks.

In this study, we demonstrated the tsRNA expression profiles during follicle selection in ducks. We found that tsRNAs mainly ranged from 27 to 32 nucleotides and the type was mostly tRF-5c in granulosa cells. Previous studies have revealed that tsRNAs can modulate gene expression at the post-transcriptional level by combining with argonaute (AGO) proteins, and tsRNAs are important regulatory factors involved in many physiological processes [26,27]. For instance, 5′tiRNA-Gly promotes muscle regeneration by targeting TGFBR1 to activate an early inflammatory response [28]. tsRNA-1599 suppresses glycolysis by increasing hexokinase 2 (HK2) expression in endothelial cells, thereby inhibiting pathological ocular angiogenesis [29]. tRNA-Gln-TTG is highly expressed in ejaculated spermatozoa and reduces the embryo cleavage rate by targeting YBX2 [30]. Herein, we performed target gene prediction and functional enrichment analyses of the top 10 differentially expressed tsRNAs. Our GO and KEGG analyses revealed the potential functions of overlapping genes in the top 10 differentially expressed tsRNA targets and differentially expressed mRNAs. Moreover, the tsRNA/mRNA/GO term interaction network revealed that multiple networks of lipid metabolism, RNA degradation, and cell cycle interactions might be regulated by tsRNAs during follicle selection in ducks.

Lipid metabolism is essential for follicle development and fecundity in poultry [31]. Increased lipid levels are a protective factor for folliculogenesis, which facilitates meiotic resumption and fertilization [32]. Recent studies have shown that de novo lipogenesis, a vital process in lipid metabolism, exists in geese at different follicle stages and promotes granulosa cell proliferation and oocyte maturation [33]. Meanwhile, non-coding RNAs, including miRNAs and lncRNAs, are involved in follicle development by modulating lipid deposition in poultry. We found that tsRNA-00764 was differentially expressed in the granulosa cells of pre-hierarchal and hierarchical follicles, and tsRNA-00764 suppressed lipid droplet accumulation in duck granulosa cells. Furthermore, steroid hormones are synthesized by granulosa cells during follicle selection in ducks [34,35]. Steroidogenesis is a multi-step process in which cholesterol is converted to active steroid hormones through several enzymatic reactions, and therefore, steroid hormone synthesis is closely related to lipid metabolism [36]. In this study, we found that tsRNA-00764 inhibited the expression of steroidogenic-related genes (STAR and CYP19A1) and reduced the concentration of estrogen and progesterone. Overall, these results indicate that tsRNA-00764 suppresses estrogen and progesterone synthesis and lipid deposition.

Peroxisome proliferator-activated receptor gamma (PPAR-γ) is a ligand-activated nuclear hormone receptor that participates in steroid synthesis, lipid deposition, inflammatory response, and autophagy [37,38,39]. Previous studies have shown that PPAR-γ is highly expressed in goose granulosa cells and differentially expressed in F1 and F5 follicles [33]. Furthermore, the expression of PPAR-γ is significantly different in the small follicles and preovulatory follicles of birds, suggesting that PPAR-γ is critical for regulating follicle development [40,41]. In this study, the transcriptomic analysis confirmed that PPAR-γ expression was higher in the hierarchical follicles than in the pre-hierarchal follicles of ducks. Additionally, our data showed that PPAR-γ promoted lipid droplet accumulation and elevated the expression of lipid deposition-related genes (FASN and LPL). Recent reports have also indicated that PPAR-γ activators and PPAR-γ overexpression improved the level of estrogen and progesterone in porcine granulosa cells and bovine granulosa cells [8]. Consistent with previous studies, we found that PPAR-γ enhanced the level of estrogen and progesterone, and increased the expression of steroidogenic-related genes (STAR and CYP19A1). In addition, we verified that PPAR-γ was the target gene of tsRNA-00764 using bioinformatics analyses and a dual-luciferase reporter gene assay, and PPAR-γ knockdown prevented the enhancement of estrogen and progesterone synthesis and lipid deposition in tsRNA-00764 inhibitor-treated cells. Taken together, our results demonstrate that tsRNA-00764 regulates estrogen and progesterone synthesis and lipid deposition by targeting PPAR-γ in duck granulosa cells.

**Figure 6:**
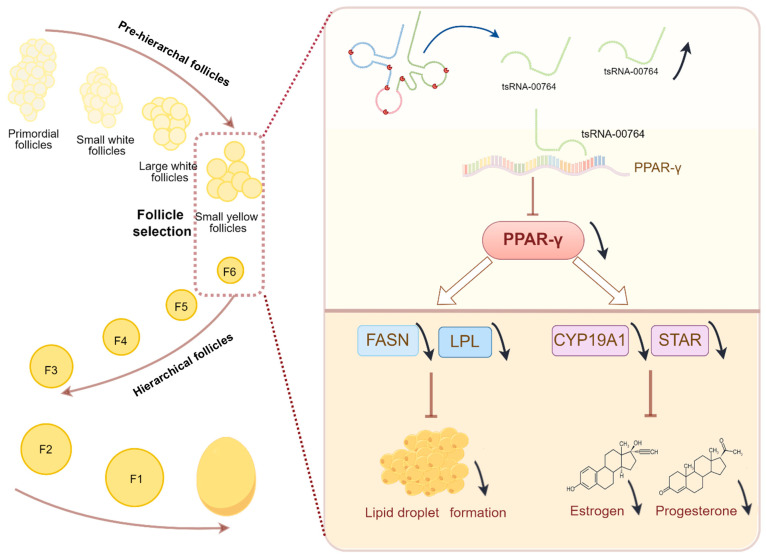
Schematic diagram of tsRNA-00764 regulatory mechanism in duck granulosa cells; estrogen and progesterone synthesis and lipid deposition are regulated by repressing PPAR-γ pathway (by Figdraw).

Overall, this study revealed the accumulation profiles of tsRNA expression by high-throughput sequencing and identified 84 differentially expressed tsRNAs between pre-hierarchal and hierarchical follicles in ducks. Specifically, the expression of tsRNA-00764 was higher in pre-hierarchal follicles than in hierarchical follicles. The combined regulatory network and enrichment analyses indicated that tsRNA-00764 had a potential regulatory role in lipid deposition during follicle selection in ducks. Furthermore, tsRNA-00764 regulated estrogen and progesterone synthesis and lipid deposition via PPAR-γ signaling in duck granulosa cells, which might affect the transition from pre-hierarchal follicles to hierarchical follicles (Figure 6). These findings will help us to better understand the role of tsRNAs in follicle development and provide novel insights to explore the underlying molecular mechanism of follicle development and production performance in ducks.

## 4. Materials and Methods

### 4.1. Animals and Sample Collection

All methods and procedures of the animal experiments were maintained in strict accordance with the Animal Care Committee of the Hubei Academy of Agricultural Sciences (Permit number: 41/2024).

A total of 10 healthy laying ducks (40 weeks) were acquired from the Poultry Breeding Department at the Institute of Animal Science and Veterinary Medicine (Wuhan, China). All ducks were housed under standardized conditions of light and temperature with food ad libitum. The ducks were humanely sacrificed in the laboratory, with the granulosa layer of each pre-hierarchal or hierarchical follicle separated from the theca layer as described previously. The granulosa cells were then stored at −80 °C [42].

### 4.2. tsRNAs-seq Library Preparation and Sequencing

RNA was extracted from granulosa cells using TRIzol (Thermo Scientific, Waltham, MA, USA), and RNA quality was assessed using Tape Station 2200 (Agilent Technologies, Santa Clara, CA, USA). We first removed RNA modifications and constructed the small RNA-seq libraries with the rtStar™ tRF and tiRNA Pretreatment Kit (AS-FS-005, Arraystar, Rockville, MD, USA). Then, tsRNA sequencing and expression quantification were, respectively, performed using NextSeq 500 (Illumina, San Diego, CA, USA) and Bioanalyzer (Agilent Technologies, California, CA, USA) at Kangcheng Biological Company (Shanghai, China). The tsRNA nomenclature was obtained from AKsomics (Aksomics Biotech, Shanghai, China).

### 4.3. Analysis of tsRNA Sequencing

The tsRNAs-seq and data analyses were performed according to the previously reported method [43]. The clean reads were harvested by trimming adaptor sequences and filtering low-quality reads. The expression profiling of tsRNAs was calculated by mapping read counts and normalizing total tsRNA reads. Differentially expressed tsRNAs were identified with fold change (|log_2_Fc| > 1) and *p*-values (*p* < 0.05) by R package DEseq2 (version 1.22.1). The tsRNA target genes were identified using TargetScan (https://www.targetscan.org/, accessed on 28 January 2024) and miRanda (http://www.microrna.org/microrna/home.do, accessed on 29 January 2024). Finally, the detailed systematic analysis of differentially expressed genes was based on GO and KEGG analyses using DAVID software (version 6.7).

### 4.4. mRNA Sequencing and Analysis

RNA sequencing was performed by Kangcheng Biological Company (Shanghai, China). The mRNA sequencing libraries were generated using the Illumina HiSeq sequencing platform, and the libraries were analyzed using the Agilent 2100 Bioanalyzer (Agilent Technologies, Santa Clara, CA, USA). Differentially expressed genes were clustered based on the RNA Adapters set1/set2 and were identified by fold change (|log_2_Fc| > 1) and *p*-values (*p*-value < 0.05).

### 4.5. Cell Culture and Transfection

Granulosa cells were separated following a previously reported method [44] and were cultured in M199 medium (11150059, Gibco, Waltham, MA, USA) containing 10% fetal bovine serum (10099141C, Gibco, Waltham, MA, USA). The amplified fragments of the coding sequences were cloned into pcDNA3.1(+). siRNAs, siRNA NC, mimics, mimic NC, inhibitors, and inhibitor NC were constructed by the RiboBio Company (Guangzhou, China). Cells were grown to 70% confluency and then transfected with plasmids, siRNAs, mimics, or inhibitors using Lipofectamine™ 8000 (C0533FT, Beyotime, Shanghai, China) or RNAiMAX (13778075, Invitrogen™, California, CA, USA). The final concentration of mimics and inhibitors in each well was 30 nM, and the siRNAs in each well came to 25 nM.

### 4.6. Luciferase Assay

The wild-type (WT) and mutant (Mut) sequence of PPAR-γ 3′UTR was cloned into the pmirGLO vector to generate PPAR-γ luciferase reporter vectors. Then, mimic NC, tsRNA mimic, pmirGLO-PPAR-γ-WT, and pmirGLO-PPAR-γ-Mut were co-transfected into granulosa cells, and a dual-luciferase assay (Promega, Madison, WI, USA) was used to determine the relative luciferase activity.

### 4.7. RNA Extraction and Quantitative RT-PCR Analyses of mRNA and tsRNA

Total RNA was harvested using an RNA extraction kit (LS1040, Promega), and the quality and concentration of RNA were determined by using the NanoDrop 2000 spectrophotometer. For mRNA analyses, RNA was reverse-transcribed to cDNA using the RevertAid RT Reverse Transcription Kit (K1691, Thermo Scientific, Waltham, MA, USA). Quantitative RT-PCR was carried out by using the Roche LightCycler 480 instrument with the Green Super Mix (172-5121, Bio-Rad, Munich, Germany). Quantification of the mRNA level was calculated with the 2^−ΔΔCt^ method after normalizing with GAPDH. The related primers are listed in Table S4.

For tsRNA analyses, RNA was reverse-transcribed to cDNA using the miScript II RT Kit (218161, Qiagen, Hilden, Germany). In a reverse transcription reaction with miScript HiFlex Buffer, tsRNA was polyadenylated by using poly(A) polymerase and converted into cDNA by using reverse transcriptase with a universal primer. Quantitative RT-PCR was carried out using the iTaqTM Universal SYBR Green Super Mix (172-5121, Bio-Rad, Hercules, CA, USA) and a Bio-Rad CFX384 system (Bio-Rad, Hercules, CA, USA) with a specific forward primer and a universal reverse primer. The procedure of quantitative RT-PCR was performed as follows: the temperature was regulated at 95 °C for 10 min, followed by 40 cycles of 95 °C for 15 s, 60 °C for 20 s, and 72 °C for 20 s. Quantification of the tsRNA level was calculated with the 2^−ΔΔCt^ method after normalizing with U6. The related primers are listed in Table S4.

### 4.8. Western Blot

The protein extracts (10–20 μg/lane) were loaded on 10% SDS gels for electrophoresis and subsequently electro-transferred onto a PVDF membrane (ISEQ00010, Millipore, Billerica, MA, USA) [45]. The transferred blots were blocked for 2 h at room temperature and incubated with primary antibodies including FASN (200194, Zenbio, Chengdu, China; 1:3000), PPAR-γ (A11183, ABclonal, Wuhan, China; 1:1000), CYP19A1 (A12684, ABclonal, Wuhan, China; 1:1000), ACTIN (AC028, ABclonal, Wuhan, China; 1:100000), LPL (R381844, Zenbio, Chengdu, China; 1:1000), STAR (A1035, ABclonal, Wuhan, China; 1:1000), and secondary antibodies. The signals were detected using the ECL Substrate Kit (170-5061, Bio-Rad, Hercules, CA, USA) and analyzed using the ChemiDocMPImaging System (Bio-Rad, Hercules, CA, USA) with ImageJ Software (version 2.0.0).

### 4.9. Oil Red O Staining of Lipid Droplet

After washing with PBS, granulosa cells were fixed with 4% paraformaldehyde at room temperature for 20 min. Granulosa cells were rinsed with isopropanol for 10 min and soaked in Oil Red O staining solution (C0157S, Beyotime) according to the manufacturer’s instructions. After three washes with PBS, the cells were stained with hematoxylin for 40 s. Images were captured with an epifluorescence microscope (Olympus BX53, Olympus, Tokyo, Japan).

### 4.10. Enzyme-Linked Immunosorbent Assay (ELISA)

The cultured duck granulosa cell supernatant was collected to measure progesterone and estrogen levels using ELISA Kits (H102-1-2 and H089-1-1, Nanjing Jiancheng Bioengineering, Nanjing, China) following the manufacturer’s instructions.

### 4.11. Statistical Analysis

All results are presented as the mean ± standard deviation. The two-group comparison was performed with a two-tailed t-test, and an independent samples t-test was used to evaluate significant differences. All experiments were performed with at least three replicates, with * *p* < 0.05, ** *p* < 0.01, and *** *p* < 0.001.

## Data Availability

The raw data supporting the conclusions of this article will be made available by the authors upon request.

## Supplementary Materials

The following supporting information can be downloaded at https://www.mdpi.com/article/10.3390/ijms252011251/s1.

## References

[1] Li D., Ning C., Zhang J., Wang Y., Tang Q., Kui H., Wang T., He M., Jin L., Li J. (2022). Dynamic Transcriptome and Chromatin Architecture in Granulosa Cells During Chicken Folliculogenesis. Nat. Commun..

[2] Han S., Wang J., Cui C., Yu C., Zhang Y., Li D., Ma M., Du H., Jiang X., Zhu Q. (2022). Fibromodulin Is Involved in Autophagy and Apoptosis of Granulosa Cells Affecting the Follicular Atresia in Chicken. Poult. Sci..

[3] Zou K., Asiamah C.A., Lu L.L., Liu Y., Pan Y., Chen T., Zhao Z., Su Y. (2020). Ovarian Transcriptomic Analysis and Follicular Development of Leizhou Black Duck. Poult. Sci..

[4] Guo Y., Zhang Y., Wang Y., Chen Q., Sun Y., Kang L., Jiang Y. (2024). Phosphorylation of Lsd1 at Serine 54 Regulates Genes Involved in Follicle Selection by Enhancing Demethylation Activity in Chicken Ovarian Granulosa Cells. Poult. Sci..

[5] Fan Y., Zhang C., Zhu G. (2019). Profiling of Rna N6-Methyladenosine Methylation During Follicle Selection in Chicken Ovary. Poult. Sci..

[6] Hao E.Y., Wang D.H., Chang L.Y., Huang C.X., Chen H., Yue Q.X., Zhou R.Y., Huang R.L. (2020). Melatonin Regulates Chicken Granulosa Cell Proliferation and Apoptosis by Activating the Mtor Signaling Pathway Via Its Receptors. Poult. Sci..

[7] Zhang X., Wei Y., Li X., Li C., Zhang L., Liu Z., Cao Y., Li W., Zhang X., Zhang J. (2022). The Corticosterone-Glucocorticoid Receptor-Ap1/Creb Axis Inhibits the Luteinizing Hormone Receptor Expression in Mouse Granulosa Cells. Int. J. Mol. Sci..

[8] Ferst J.G., Rovani M.T., Dau A.M.P., Gasperin B.G., Antoniazzi A.Q., Bordignon V., Oliveira D.E., Gonçalves P.B.D., Ferreira R. (2020). Activation of Pparg Inhibits Dominant Follicle Development in Cattle. Theriogenology.

[9] Chen X., Huang K., Hu S., Lan G., Gan X., Gao S., Deng Y., Hu J., Li L., Hu B. (2022). Integrated Transcriptome and Metabolome Analysis Reveals the Regulatory Mechanisms of Fasn in Geese Granulosa Cells. Int. J. Mol. Sci..

[10] Cui Z., Ning Z., Deng X., Du X., Amevor F.K., Liu L., Kang X., Tian Y., Wang Y., Li D. (2022). Integrated Proteomic and Metabolomic Analyses of Chicken Ovary Revealed the Crucial Role of Lipoprotein Lipase on Lipid Metabolism and Steroidogenesis During Sexual Maturity. Front. Physiol..

[11] Xu Z., Liu Q., Ning C., Yang M., Zhu Q., Li D., Wang T., Li F. (2024). Mirna Profiling of Chicken Follicles During Follicular Development. Sci. Rep..

[12] Huang Y., Li S., Tan Y., Xu C., Huang X., Yin Z. (2024). Identification and Functional Analysis of Ovarian Lncrnas During Different Egg Laying Periods in Taihe Black-Bone Chickens. Front. Physiol..

[13] Meng L., Teerds K., Tao J., Wei H., Jaklofsky M., Zhao Z., Liang Y., Li L., Wang C.C., Zhang S. (2020). Characteristics of Circular Rna Expression Profiles of Porcine Granulosa Cells in Healthy and Atretic Antral Follicles. Int. J. Mol. Sci..

[14] Deng X., Ning Z., Li L., Cui Z., Du X., Amevor F.K., Tian Y., Shu G., Du X., Han X. (2023). High Expression of Mir-22-3p in Chicken Hierarchical Follicles Promotes Granulosa Cell Proliferation, Steroidogenesis, and Lipid Metabolism Via Pten/Pi3k/Akt/Mtor Signaling Pathway. Int. J. Biol. Macromol..

[15] Wu Y., Xiao H., Pi J., Zhang H., Pan A., Pu Y., Liang Z., Shen J., Du J., Huang T. (2021). Lncrna Lnc_13814 Promotes the Cells Apoptosis in Granulosa Cells of Duck by Acting as Apla-Mir-145-4 Sponge. Cell Cycle.

[16] He H., Wei Y., Chen Y., Zhao X., Shen X., Zhu Q., Yin H. (2024). High Expression Circralgps2 in Atretic Follicle Induces Chicken Granulosa Cell Apoptosis and Autophagy Via Encoding a New Protein. J. Anim. Sci. Biotechnol..

[17] Chen Q., Li D., Jiang L., Wu Y., Yuan H., Shi G., Liu F., Wu P., Jiang K. (2024). Biological Functions and Clinical Significance of Trna-Derived Small Fragment (Tsrna) in Tumors: Current State and Future Perspectives. Cancer Lett..

[18] Xiong Q., Zhang Y. (2023). Small Rna Modifications: Regulatory Molecules and Potential Applications. J. Hematol. Oncol..

[19] Zhao Y., Li X., Ye C., Huang C., Lv X., Li J. (2023). The Biogenesis, Mechanism and Function of the Trna-Derived Small Rna (Tsrna): A Review Compared with Microrna. Am. J. Cancer Res..

[20] Liu J.G., Xia W.G., Chen W., Abouelezz K.F.M., Ruan D., Wang S., Zhang Y.N., Huang X.B., Li K.C., Zheng C.T. (2021). Effects of Capsaicin on Laying Performance, Follicle Development, and Ovarian Antioxidant Capacity in Aged Laying Ducks. Poult. Sci..

[21] Zhao J., Pan H., Zhao W., Li W., Li H., Tian Z., Meng D., Teng Y., Li X., He Y. (2023). Untargeted Metabolomics Revealed Potential Biomarkers of Small Yellow Follicles of Chickens During Sexual Maturation. Metabolites.

[22] Chen W., Xia W.G., Ruan D., Wang S., Abouelezz K.F.M., Wang S.L., Zhang Y.N., Zheng C.T. (2020). Dietary Calcium Deficiency Suppresses Follicle Selection in Laying Ducks through Mechanism Involving Cyclic Adenosine Monophosphate-Mediated Signaling Pathway. Animal.

[23] Cai J., Li C., Liu S., Tan M., Sun Y., Sun X., Yang M., He B. (2024). Angiogenin-Mediated Tsrnas Control Inflammation and Metabolic Disorder by Regulating Nlrp3 Inflammasome. Cell Death Differ..

[24] Zhang Y., Labrecque R., Tremblay P., Plessis C., Dufour P., Martin H., Sirard M.A. (2024). Sperm-Borne Tsrnas and Mirnas Analysis in Relation to Dairy Cattle Fertility. Theriogenology.

[25] Liao T., Gan M., Lei Y., Wang Y., Chen L., Shen L., Zhu L. (2023). Dynamic Changes in the Transcriptome of Trna-Derived Small Rnas Related with Fat Metabolism. Sci. Data.

[26] Zhang S., Gu Y., Ge J., Xie Y., Yu X., Wu X., Sun D., Zhang X., Guo J., Guo J. (2024). Trf-33-P4r8yp9lon4vdp Inhibits Gastric Cancer Progression Via Modulating Stat3 Signaling Pathway in an Ago2-Dependent Manner. Oncogene.

[27] Di Fazio A., Gullerova M. (2023). An Old Friend with a New Face: Trna-Derived Small Rnas with Big Regulatory Potential in Cancer Biology. Br. J. Cancer.

[28] Shen L., Liao T., Chen Q., Lei Y., Wang L., Gu H., Qiu Y., Zheng T., Yang Y., Wei C. (2023). Trna-Derived Small Rna, 5′tirna-Gly-Ccc, Promotes Skeletal Muscle Regeneration through the Inflammatory Response. J. Cachexia Sarcopenia Muscle.

[29] Han X.Y., Kong L.J., Li D., Tong M., Li X.M., Zhao C., Jiang Q., Yan B. (2024). Targeting Endothelial Glycolytic Reprogramming by Tsrna-1599 for Ocular Anti-Angiogenesis Therapy. Theranostics.

[30] Chen X., Zheng Y., Lei A., Zhang H., Niu H., Li X., Zhang P., Liao M., Lv Y., Zhu Z. (2020). Early Cleavage of Preimplantation Embryos Is Regulated by Trna(Gln-Ttg)-Derived Small Rnas Present in Mature Spermatozoa. J. Biol. Chem..

[31] Zhang L., Wang E., Peng G., Wang Y., Huang F. (2023). Comprehensive Proteome and Acetyl-Proteome Atlas Reveals Hepatic Lipid Metabolism in Layer Hens with Fatty Liver Hemorrhagic Syndrome. Int. J. Mol. Sci..

[32] Liu T., Qu J., Tian M., Yang R., Song X., Li R., Yan J., Qiao J. (2022). Lipid Metabolic Process Involved in Oocyte Maturation During Folliculogenesis. Front. Cell Dev. Biol..

[33] Wen R., Gan X., Hu S., Gao S., Deng Y., Qiu J., Sun W., Li L., Han C., Hu J. (2019). Evidence for the Existence of De Novo Lipogenesis in Goose Granulosa Cells. Poult. Sci..

[34] Yang C., Luo P., Yang Y.T., Fu X.L., Li B.X., Shen X., Xu D.N., Huang Y.M., Tian Y.B., Liu W.J. (2024). Drp1 Regulated Pink1-Dependent Mitophagy Protected Duck Follicular Granulosa Cells from Acute Heat Stress Injury. Poult. Sci..

[35] Luo P., Huang X.B., Zhan X.Z., Yang C., Deng Z.C., Zhang C., Fu X.L., Tian Y.B., Huang Y.M., Liu W.J. (2023). Heat Enhances the Inhibitory Effect of Lipopolysaccharide on Duck Granulosa Cell Proliferation and Steroid Biosynthesis in Vitro. Anim. Sci. J..

[36] Kolaitis N.D., Finger B.J., Merriner D.J., Nguyen J., Houston B.J., O’Bryan M.K., Stringer J.M., Zerafa N., Nguyen N., Hutt K.J. (2023). Impact of Chronic Multi-Generational Exposure to an Environmentally Relevant Atrazine Concentration on Testicular Development and Function in Mice. Cells.

[37] Ning Z., Deng X., Li L., Feng J., Du X., Amevor F.K., Tian Y., Li L., Rao Y., Yi Z. (2023). Mir-128-3p Regulates Chicken Granulosa Cell Function Via 14-3-3β/Foxo and Ppar-Γ/Lpl Signaling Pathways. Int. J. Biol. Macromol..

[38] Zhang B., Zeng M., Wang Y., Li M., Wu Y., Xu R., Zhang Q., Jia J., Huang Y., Zheng X. (2022). Oleic Acid Alleviates Lps-Induced Acute Kidney Injury by Restraining Inflammation and Oxidative Stress Via the Ras/MAPKs/PPAR-γ Signaling Pathway. Phytomedicine.

[39] Zhong C.C., Zhao T., Hogstrand C., Chen F., Song C.C., Luo Z. (2022). Copper (Cu) Induced Changes of Lipid Metabolism through Oxidative Stress-Mediated Autophagy and Nrf2/Pparγ Pathways. J. Nutr. Biochem..

[40] Wu H., Yuan J., Yin H., Jing B., Sun C., Nguepi Tsopmejio I.S., Jin Z., Song H. (2023). Flammulina Velutipes Stem Regulates Oxidative Damage and Synthesis of Yolk Precursors in Aging Laying Hens by Regulating the Liver-Blood-Ovary Axis. Poult. Sci..

[41] Li Q., Hu S., Wang Y., Deng Y., Yang S., Hu J., Li L., Wang J. (2019). Mrna and Mirna Transcriptome Profiling of Granulosa and Theca Layers from Geese Ovarian Follicles Reveals the Crucial Pathways and Interaction Networks for Regulation of Follicle Selection. Front. Genet..

[42] Ran M., Hu S., Ouyang Q., Xie H., Zhang X., Lin Y., Li X., Hu J., Li L., He H. (2023). miR-202-5p Inhibits Lipid Metabolism and Steroidogenesis of Goose Hierarchical Granulosa Cells by Targeting ACSL3. Animals.

[43] Zhao J.Z., Li Q.Y., Lin J.J., Yang L.Y., Du M.Y., Wang Y., Liu K.X., Jiang Z.A., Li H.H., Wang S.F. (2022). Integrated Analysis of Trna-Derived Small Rnas in Proliferative Human Aortic Smooth Muscle Cells. Cell Mol. Biol. Lett..

[44] Wu Y., Xiao H., Pi J., Zhang H., Pan A., Pu Y., Liang Z., Shen J., Du J. (2020). The Circular Rna Aplacirc_13267 Upregulates Duck Granulosa Cell Apoptosis by the Apla-Mir-1-13/Thbs1 Signaling Pathway. J. Cell Physiol..

[45] Sonowal R., Swimm A.I., Cingolani F., Parulekar N., Cleverley T.L., Sahoo A., Ranawade A., Chaudhuri D., Mocarski E.S., Koehler H. (2023). A Microbiota and Dietary Metabolite Integrates DNA Repair and Cell Death to Regulate Embryo Viability and Aneuploidy During Aging. Sci. Adv..

